# BAG6 regulates the quality control of a polytopic ERAD substrate

**DOI:** 10.1242/jcs.145565

**Published:** 2014-07-01

**Authors:** Aishwarya Payapilly, Stephen High

**Affiliations:** Faculty of Life Sciences, The University of Manchester, Michael Smith Building, Oxford Road, Manchester M13 9PT, UK

**Keywords:** Degradation, Dominant-negative, Endoplasmic-reticulum-associated degradation, Proteasome, Stabilisation, Ubiquitin

## Abstract

BAG6 participates in protein quality control and, here, we address its role in endoplasmic-reticulum-associated degradation (ERAD) by using the polytopic membrane protein OpD, an opsin degron mutant. Both BAG6 knockdown and BAG6 overexpression delay OpD degradation; however, our data suggest that these two perturbations are mechanistically distinct. Hence, BAG6 knockdown correlates with reduced OpD polyubiquitylation, whereas BAG6 overexpression increases the level of polyubiquitylated OpD. The UBL- and BAG-domains of exogenous BAG6 are dispensable for OpD stabilisation and enhanced levels of polyubiquitylated OpD. Thus, although endogenous BAG6 normally promotes OpD degradation, exogenous BAG6 expression delays this process. We speculate that overexpressed BAG6 subunits might associate with the endogenous BAG6 complex, resulting in a dominant-negative effect that inhibits its function. Interestingly, cellular levels of BAG6 also correlate with total steady-state polyubiquitylation, with Rpn10 (officially known as PSMD4) overexpression showing a similar effect. These findings suggest that perturbations of the levels of ubiquitin-binding proteins can impact upon cellular ubiquitin homeostasis. We propose that exogenous BAG6 perturbs the function of the BAG6 complex at a stage subsequent to substrate recognition and polyubiquitylation, most likely the BAG6-dependent delivery of OpD to the proteasome.

## INTRODUCTION

Up to one third of all proteins synthesised in the eukaryotic cytosol are predicted to be translocated into the endoplasmic reticulum (ER) in order to undergo secretion or membrane insertion (Ghaemmaghami et al., 2003). The ER is equipped with machinery that facilitates the appropriate folding, co- and post-translational modification and assembly of these proteins, enabling them to attain their final native conformations (Ghaemmaghami et al., 2003; Jonikas et al., 2009; Lynes and Simmen, 2011). However, protein folding and assembly can be inefficient, resulting in substantial amounts of misfolded and potentially toxic proteins that are unable to attain their native conformation. Hence, there is a need for a triage system at the ER to continuously assess the folding status of newly synthesised polypeptides, distinguish between transiently unfolded and terminally misfolded proteins and deal with them accordingly (Buchberger et al., 2010; Goeckeler and Brodsky, 2010). Aberrant proteins are typically removed from the ER in a process called ER-associated degradation (ERAD) (Claessen et al., 2012). ERAD involves the coordinated actions of various chaperones, enzymes and co-factors located at the ER, which recognise misfolded protein substrates and promote their retrotranslocation into the cytoplasm for degradation by the ubiquitin-mediated proteasomal pathway.

The BAG6 protein is composed of a proline-rich central region flanked by an N-terminal ubiquitin-like (UBL) domain and a C-terminal Bcl-2-associated anthanogene (BAG) domain (cf. Leznicki et al., 2013). It forms part of a complex containing BAG6, TRC35 (also known as GET4) and UBL4A (Kawahara et al., 2013) that was recently implicated in promoting the ERAD of a soluble variant of ribophorin (RI_332_) and the single-spanning membrane proteins TCRα, CD4 and human major histocompatibility complex (MHC) class I heavy chain (Claessen and Ploegh, 2011; Ernst et al., 2011; Wang et al., 2011). The actions of the BAG6 complex have been linked to both derlin2 (Claessen and Ploegh, 2011) and the p97 retrotranslocation complex, in the latter case through interactions with ER-membrane-localised proteins, including HRD1, gp78 and UbXD8 (also known as SYVN1, AMFR and FAF2, respectively) (Wang et al., 2011; Xu et al., 2013). Current models suggest that the BAG6 complex promotes ERAD by acting as a ‘holdase’ to maintain the solubility of aggregation-prone misfolded proteins located in the cytosol (Kawahara et al., 2013; Lee and Ye, 2013; Wang et al., 2011). The UBL domain of BAG6 can interact with the Rpn10 subunit (officially known as PSMD4) of the 26S proteasome and, thus, it most likely provides a platform for both protein polyubiquitylation and the transfer of substrates to the ubiquitin proteasome system (UPS) for degradation (Hessa et al., 2011; Kikukawa et al., 2005; Minami et al., 2010). Links between the BAG6 complex and the UPS are further underlined by its recently identified role in enabling proteasome assembly (Akahane et al., 2013).

The ability of the BAG6 complex to recognise and bind to exposed hydrophobic domains of misfolded proteins, such as ERAD substrates, has been suggested to be mediated by the central disordered proline-rich region of its BAG6 subunit, which binds to tail-anchored protein substrates (Leznicki et al., 2013). This region of the BAG6 protein was also shown to be required for maintaining the solubility of denatured luciferase and for the ability of the trimeric BAG6 complex to form higher-order homo-oligomers (Xu et al., 2013). The resulting BAG6 complex oligomers have the potential to interact simultaneously with multiple facilitators of ERAD and the UPS, thereby providing a basis for coordinating the transfer of substrates between them.

The BAG6 subunit is a crucial component of the BAG6–TRC35–UBL4A complex and, hence, a BAG6 knockdown results in reduced levels of endogenous TRC35 and UBL4A, but not vice versa (Krenciute et al., 2013). The UBL domains of the UBL4A and/or BAG6 subunits of the complex can also recruit SGTA, a known BAG6 binding partner that modulates its quality control functions both *in vitro* and *in vivo* (Leznicki and High, 2012; Leznicki et al., 2013; Xu et al., 2012). The BAG6 protein also has a well-defined bi-partite nuclear localisation signal (NLS) (Manchen and Hubberstey, 2001), and it is the TRC35 subunit that appears to be responsible for maintaining a cytosolic pool of the trimeric BAG6 complex (Wang et al., 2011). The presence of a functional NLS enables BAG6 to shuttle between the cytosol and the nucleus in a cell-cycle-dependent manner (Yong and Wang, 2012) and thereby to function at both cellular locations. In addition to its role in protein quality control, BAG6 can influence a diverse range of cellular processes, including cell cycle regulation, DNA damage repair, apoptosis, membrane protein translocation, T-cell response, antigen presentation and signal transduction (Kawahara et al., 2013; Lee and Ye, 2013).

In this study, we establish that an aberrant seven-transmembrane-domain protein, the opsin degron mutant (OpD) (Ray-Sinha et al., 2009), is a BAG6-dependent substrate for ERAD. We identify a novel dominant-negative effect of BAG6 overexpression on OpD degradation and exploit this effect to gain insights into BAG6 function, focusing on the contributions of its UBL and BAG domains. Interestingly, we find that BAG6 not only regulates the polyubiquitylation of the aberrant membrane protein OpD, but it also influences the total cellular level of polyubiquitylated proteins, irrespective of ERAD substrate production. This effect is recapitulated by a second ubiquitin-binding protein, Rpn10, and our data suggest that the influence of the BAG6 complex on global ubiquitin homeostasis might contribute to some of the diverse cellular processes in which it has previously been implicated. In the context of protein quality control, our data support a model whereby the BAG6 complex plays a pivotal role in promoting both the polyubiquitylation of ERAD substrates and their delivery to the proteasome.

## RESULTS

### A multi-transmembrane domain ERAD substrate requires BAG6 for efficient degradation

**Fig. 1.**
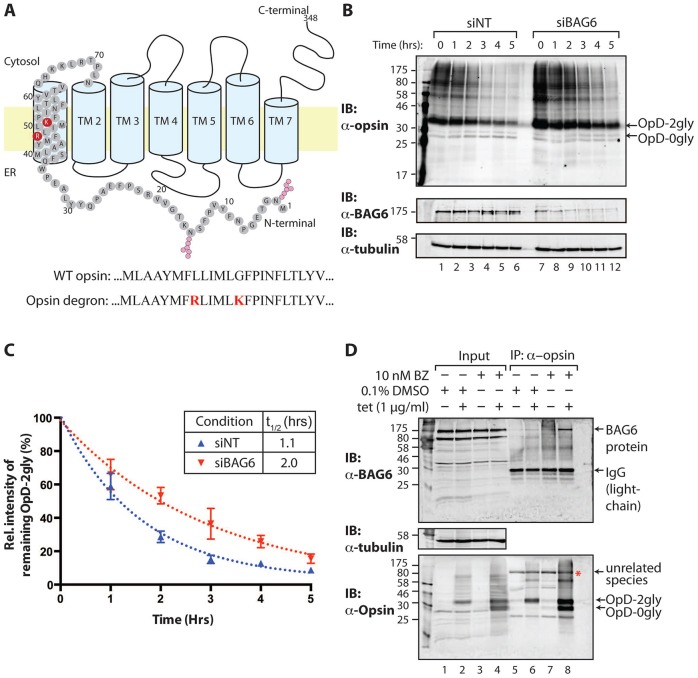
**BAG6 plays a direct role in OpD ERAD.** (A) Diagram showing the ER membrane topology of OpD, depicting N-glycosylation sites (pink) at residues 2 and 15 and mutated residues (red) in transmembrane domain (TM)1. Partial sequences of TM1 in wild-type opsin and the opsin degron mutant highlight the degron motif in OpD (red). (B) A 5-hour cycloheximide chase was performed using stable inducible HeLa-OpD cells that were first transfected with either control non-targeting RNA (siNT, lanes 1–6) or RNA targeted against BAG6 (siBAG6, lanes 7–12). After 48 hours, cells were induced to express OpD for a further 16–20 hours and were then either harvested directly into sample buffer (0 hour) or treated with cycloheximide to inhibit further protein synthesis. Whole-cell lysates were subsequently prepared at 1-hour intervals and a fraction of these samples (5%) was analysed by immunoblotting to detect OpD, endogenous BAG6 and tubulin. 2gly, di-glycosylated; 0gly, non-glycosylated. (C) OpD levels were quantified at each time-point, and the resulting data was analysed as described in Materials and Methods. OpD levels are shown as the mean±s.e.m. (at least three independent repeats). Curves of best fit for each condition are also shown. (D) Interaction between OpD and endogenous BAG6. HeLa OpD cells were induced to express OpD for 24 hours and were simultaneously treated overnight with either 0.1% (v/v) DMSO (lanes 1, 2, 5 and 6) or 10 nM bortezomib (BZ, lanes 3, 4, 7 and 8). Cells were lysed using a digitonin-containing buffer, and OpD was immunoprecipitated. Immunoblotting (IB) of total cell lysates (lanes 1–4) and immunoprecipitated (IP) material (lanes 5–8) showed that BAG6 was co-immunoprecipitated with OpD upon proteasomal inhibition (lane 8). Red asterisk, a non-related species most likely recognised by the primary antibodies used for immunoprecipitation.

OpD is a previously established ERAD substrate that was derived from wild-type opsin by the introduction of a degron motif into the first transmembrane domain (Fig. 1A). OpD is known to undergo proteasomal degradation following ubiquitylation by the HRD1 E3 ligase (Ray-Sinha et al., 2009). To assess whether the ERAD of the multi-spanning membrane protein OpD was facilitated by BAG6, endogenous levels of BAG6 were reduced by siRNA treatment as described previously (Winnefeld et al., 2006), using a stable inducible OpD-expressing HeLa cell line. Following a suitable period of siRNA-mediated knockdown, resulting in a ∼85% reduction in the expression of endogenous BAG6, cells were induced to express OpD, after which a cycloheximide chase was performed to study the stability of pre-existing OpD in the absence of on-going protein synthesis (Fleig et al., 2012; Yewdell et al., 2011). The relative amount of OpD present in SDS-solubilised whole-cell lysates at each time-point was then determined by quantitative immunoblotting (see Materials and Methods). In control cells, the levels of OpD decreased following the addition of cycloheximide, with an estimated half-life of ∼1.1 hours, consistent with its removal through ERAD (Ray-Sinha et al., 2009). Upon BAG6 knockdown, OpD showed an increased half-life of ∼2 hours (Fig. 1B, IB: α-opsin; Fig. 1C), thereby suggesting that endogenous BAG6 promotes the degradation of this model ERAD substrate.

To assess whether BAG6 plays a direct role in promoting OpD degradation, we tested for an interaction between the two components following solubilisation of cells with a digitonin-containing buffer, chosen to preserve protein–protein interactions (Wiertz et al., 1996b). Interactions between misfolded proteins and components that promote their degradation are often labile or transient and can best be captured when degradation is inhibited (Wang et al., 2011). For this reason, we carried out this analysis in both the presence and absence of bortezomib, a proteasome inhibitor that we found to delay OpD degradation, resulting in the accumulation of a cytosolic deglycosylated form of the protein that is otherwise rapidly degraded (supplementary material Fig. S1A,B,D). Bortezomib treatment followed by immunoprecipitation of OpD revealed a robust association of the protein with endogenous BAG6, which was not detected in a DMSO-treated control sample (Fig. 1D, IB: α-BAG6, cf. lanes 6, 8). This is consistent with a direct interaction between OpD and the BAG6 complex, and our data also suggest that BAG6 might preferentially interact with a deglycosylated cytosolic form of this novel ERAD substrate (Fig. 1D, IB: α-opsin, cf. lanes 6, 8), which is most likely cytosolic (Wiertz et al., 1996a).

**Fig. 2.**
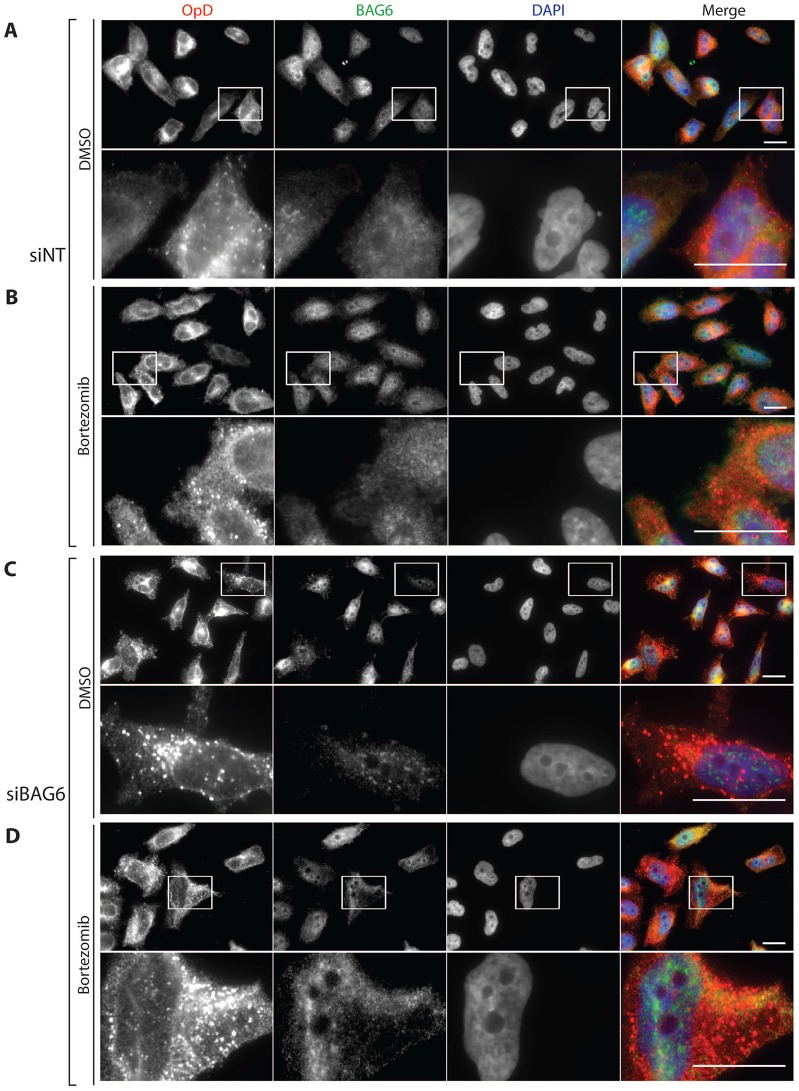
**BAG6 knockdown increases the number of OpD-positive punctae.** OpD HeLa cells were transfected with either control non-targeting siRNA (siNT; A,B) or BAG6-targeting siRNA (siBAG6; C,D) and, 48 hours later, OpD expression was induced for an additional 16–20 hours. Cells were treated with either 0.1% (v/v) DMSO (A,C) or 10 µM bortezomib (B,D) for 5 hours, before being fixed and immunostained with anti-opsin (red) and anti-BAG6 (green) antibodies. Nuclei were stained with DAPI (blue). Specific regions of interest within a field are indicated by white boxes and are shown in magnified form below the images they were taken from. Scale bars: 10 µm.

In order to further explore the effect of reducing endogenous BAG6 levels on the fate of OpD, immunofluorescence microscopy was performed. In control HeLa OpD cells (treated with a non-targeting siRNA), a monoclonal antibody against the opsin N-terminus labelled extensive regions of the cell, consistent with an ER localisation (Fig. 2A) that might also extend to the ER–Golgi intermediate compartment (Ray-Sinha et al., 2009). These control cells also displayed several OpD-positive punctae that appeared to be enhanced upon treatment with the proteasome inhibitor bortezomib (Fig. 2B), suggesting that they might result from the inefficient clearance of OpD. At a qualitative level, the number and distribution of these punctae also appeared to increase following a BAG6 knockdown (Fig. 2C), with the effects of BAG6 knockdown and bortezomib treatment being potentially additive (Fig. 2D). A comparable effect on the level of TCRα-positive punctae has been observed previously upon BAG6 knockdown, and the authors concluded that these structures contain aggregated TCRα (Wang et al., 2011). Further analysis will be required to determine the nature of the OpD-positive punctae that we observe here.

### Overexpressing the BAG6 subunit inhibits ERAD of OpD

**Fig. 3.**
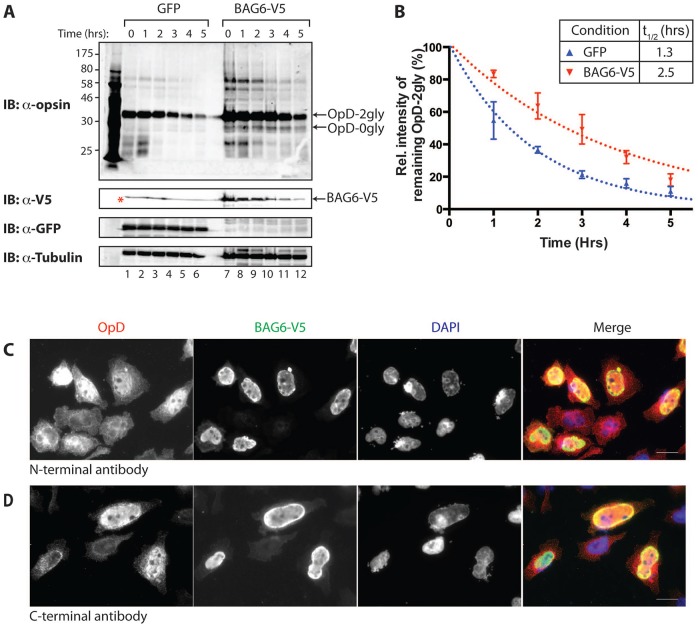
**Exogenous expression of BAG6 inhibits OpD degradation and enables its nuclear relocalisation.** (A) GFP or BAG6–V5 were transiently expressed for 24 hours in stable HeLa-OpD cells before the expression of OpD was induced for an additional 16–20 hours. The degradation of OpD in the presence of GFP or BAG6–V5 was compared by immunoblot (IB) analysis of whole-cell lysates prepared at 0 hours and at 1-hour intervals during a 5-hour cycloheximide chase. Red asterisk, cross-reacting species of unknown origin observed in control samples (lanes 1–6) and in an empty lane (between lanes 6 and 7). 2gly, di-glycosylated; 0gly, non-glycosylated. (B) OpD levels were quantified at each time-point, and the resulting data were analysed as described in Materials and Methods. OpD levels are shown as the mean±s.e.m. (at least three independent repeats). Curves of best fit for each condition are also shown. (C,D) The effect of BAG6–V5 overexpression on OpD was visualised by immunofluorescence microscopy. Stable HeLa-OpD cells transiently expressing BAG6–V5 for 24 hours were induced to express OpD for an additional 16–20 hours before being fixed and stained with anti-opsin antibodies to detect the N- and C-termini of OpD (red, as indicated) and anti-V5 antibody to detect overexpressed BAG6 (green). DNA is stained with DAPI (blue). Scale bars: 10 µm.

To gain further insight into the role of BAG6 during the quality control of OpD, a V5-tagged version of BAG6 isoform 2 (BAG6–V5) was transiently expressed in HeLa OpD cells, and OpD degradation was analysed using cycloheximide chase and quantitative immunoblotting of whole-cell lysates as outlined above. At a biochemical level, the effect of exogenous BAG6 expression was directly comparable to that of siRNA-mediated BAG6 depletion; hence, the half-life of OpD in the presence of exogenous BAG6 was ∼2.5 hours, but only ∼1.3 hours in control cells expressing GFP (Fig. 3A,B). Furthermore, the effect of exogenous BAG6–V5 on OpD degradation was comparable to that of bortezomib treatment (supplementary material Fig. S1C) and appeared to correlate with the expression level of the exogenous protein (supplementary material Fig. S1D). This apparently ‘dominant-negative’ effect of expressing a tagged form of the wild-type protein might reflect the fact that BAG6 normally functions as part of an oligomeric complex (Prelich, 2012; see Discussion).

Interestingly, although the increase in the half-life of OpD was comparable following BAG6–V5 overexpression and BAG6 knockdown (Fig. 1C; Fig. 3B), immunofluorescence microscopy suggests that the mechanistic basis of these two perturbations are distinct. Hence, despite the stabilising effect of BAG6 overexpression on OpD, we did not observe any evidence for the qualitative increase in cytosolic OpD-positive punctae that accompanied a BAG6 knockdown (Fig. 3C; Fig. 2C). Furthermore, immunofluorescence studies of cells expressing exogenous BAG6 showed that a substantial amount of OpD relocalised to the nucleus (Fig. 3C). This non-physiological nuclear relocalisation phenotype mirrors an effect that has been observed when BAG6 is expressed in yeast (Leznicki et al., 2010) and is indicative of a direct association between exogenous BAG6–V5 and OpD. Intriguingly, monoclonal antibodies recognising both the N- and C-termini of the substrate (Fig. 3C,D, respectively) each detected OpD in the nucleus of cells coexpressing exogenous BAG6–V5. This indicates that both termini of our model ERAD substrate are present in this nuclear OpD population, raising the possibility that full-length intact OpD is relocalised to the nucleus upon BAG6 overexpression.

**Fig. 4.**
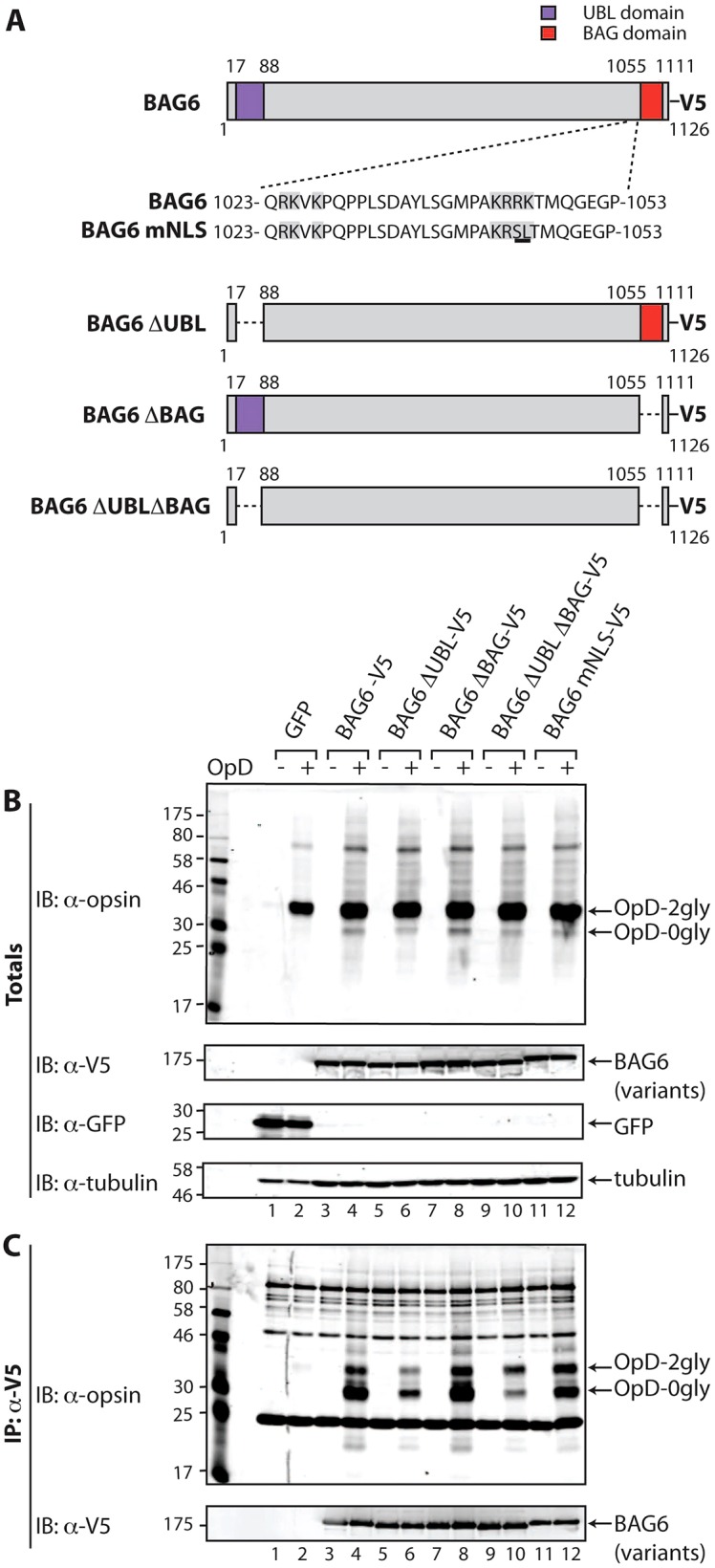
**The UBL domain of BAG6 promotes its association with OpD.** (A) Illustration of the V5-tagged deletion mutants of BAG6 (isoform 2) generated for this study. (B,C) Interactions of OpD with different forms of BAG6 were compared by overexpressing the V5-tagged BAG6 variants for 24 hours, followed by induction of OpD expression for a further 16–20 hours and then the immunoprecipitation of the BAG6 variants and any associated OpD from cell lysates prepared using a digitonin-containing buffer (cf. Fig. 1D). (B) 5% of the samples were kept aside as input controls (totals) and were immunoblotted (IB) with antibodies to detect OpD, V5-tagged BAG6 variants, GFP and tubulin. (C) Proteins that immunoprecipitated (IP) with the anti-V5 antibody were eluted from Protein-A–Sepharose, and the resulting samples were probed for OpD and V5-tagged BAG6 variants by immunoblotting. 2gly, di-glycosylated; 0gly, non-glycosylated.

To test whether nuclear relocalisation contributed to the stabilising effect of exogenous BAG6 expression, for example by sequestering a fraction of OpD in the nucleus where it would be protected from cytoplasmic degradation, a mutant version of BAG6 that could not undergo nuclear import (BAG6 mNLS, Fig. 4A) was exploited. Although the expression of BAG6-mNLS–V5 prevented any detectable nuclear relocalisation of OpD (supplementary material Fig. S2A), it continued to inhibit the degradation of OpD at a level comparable to that seen in the BAG6–V5-overexpressing cells (supplementary material Fig. S2B,C). We therefore conclude that the relocalisation of the substrate to the nucleus is not a key factor for the inhibition of OpD degradation that is observed upon exogenous BAG6 expression.

### The UBL domain is required for efficient substrate loading onto BAG6

Having established that the expression of BAG6–V5 resulted in an inhibition of OpD degradation, we used this strategy to investigate the contribution of its characteristic UBL and BAG domains (Fig. 4A; Leznicki et al., 2013). Several lines of evidence suggest a direct interaction between the BAG6 subunit and a range of hydrophobic substrates (Kawahara et al., 2013; Leznicki et al., 2010; Leznicki et al., 2013), consistent with the association of OpD with the endogenous BAG6 complex (Fig. 1D) and the relocalisation of OpD to the nucleus observed upon coexpression with BAG6–V5 (Fig. 3C,D). On this basis, we first asked whether we could observe a direct physical association between exogenous BAG6–V5 and OpD and, if so, whether this was influenced by the UBL or BAG domains. When OpD expression was induced in the presence of BAG6–V5, we observed a robust and specific co-immunoprecipitation of these two components (Fig. 4B,C, cf. lanes 2, 4). Notably, although the deglycosylated form of OpD (OpD-0gly) is a minor component of the total cell lysate (Fig. 4B, IB: α-opsin, lanes 2, 4), this was the predominant species in the BAG6–V5-associated OpD material (Fig. 4C, see IB: α-opsin, lane 4). This is consistent with exogenous BAG6 recognising OpD as a hydrophobic substrate, and it suggests that BAG6–V5 most likely associates with OpD during, or shortly after, its retrotranslocation (see Discussion). Furthermore, the ability of BAG6–V5 to bind to OpD suggests that the inclusion of a short C-terminal tag does not abolish BAG6 function, although we cannot formally exclude the possibility that it has some effect.

BAG6 has been suggested to have a ‘holdase’ activity (Wang et al., 2011) and, in order to rule out the possibility that the observed association with OpD occurs post-lysis, we carried out an additional control experiment. Exogenous BAG6–V5 and OpD were individually expressed, the host cells were lysed separately and the resulting digitonin extracts were then mixed and processed as before. Although both components are individually expressed at levels comparable to those seen when coexpressed (Fig. 4), we observed no association of the two components under these conditions (supplementary material Fig. S3B). We therefore concluded that the observed co-immunoprecipitation of BAG6 and OpD reflects a bona fide interaction, and we proceeded to analyse selected BAG6 mutants. We generated mutant forms of BAG6–V5 lacking either the N-terminal UBL domain (BAG6-ΔUBL–V5), the C-terminal BAG domain (BAG6-ΔBAG–V5) or both (BAG6-ΔUBLΔBAG–V5) (Fig. 4A), and we analysed their association with OpD as before. Although the expression and immunoprecipitation of BAG6–V5 and its mutants were comparable (Fig. 4B,C, see IB: α-V5, lanes 3–12), their ability to co-immunoprecipitate OpD was quite distinct. Hence, the behaviour of the BAG6-ΔBAG–V5 mutant was almost identical to that of the full-length protein, and we observed a robust co-immunoprecipitation, with an enrichment of the deglycosylated form of OpD in the associated material (Fig. 4C, IB: α-opsin, lanes 4, 8). A similar pattern was also observed with the BAG6-mNLS–V5 version (Fig. 4A; supplementary material Fig. S2), indicating that the ability of exogenous BAG6 to enter the nucleus does not affect its co-immunoprecipitation with OpD (Fig. 4C, IB: α-opsin). By contrast, the loss of the UBL domain, either alone (BAG6-ΔUBL–V5) or in combination with the BAG domain (BAG6-ΔUBLΔBAG–V5), resulted in a substantial reduction, although not a complete loss, of OpD that was co-immunoprecipitated with BAG6 (Fig. 4C, IB: α-opsin, cf. lanes 4, 6, 10). Taken together, these data suggest that the UBL domain of BAG6 is important for efficient substrate recruitment.

Having investigated the role of the UBL and BAG domains in the association of exogenous BAG6 with the model ERAD substrate OpD, we next addressed the contribution of these regions to the inhibitory effect of exogenous BAG6–V5 expression on OpD degradation. To this end, we induced OpD expression in cells transiently expressing BAG6–V5 or its mutant forms and analysed the half-life of OpD degradation having imposed a cycloheximide block (Fig. 1; supplementary material Fig. S4). In marked contrast to the effect of UBL deletion upon the association of BAG6 with OpD (Fig. 4), loss of the UBL domain, the BAG domain or both did not significantly alter the prolonged half-life of OpD that was observed in the presence of BAG6–V5 (supplementary material Fig. S4).

### BAG6 affects steady-state polyubiquitylation

Although it has been suggested that BAG6 can promote substrate polyubiquitylation (Minami et al., 2010), a knockdown of BAG6 has been found to lead to an accumulation of polyubiquitylated TCRα, a well-defined ERAD substrate (Wang et al., 2011). Given this potential complexity, and our finding that BAG6 levels influence OpD degradation (Figs 1, 3), we next investigated the polyubiquitylation status of our model ERAD substrate following perturbation of cellular BAG6.

**Fig. 5.**
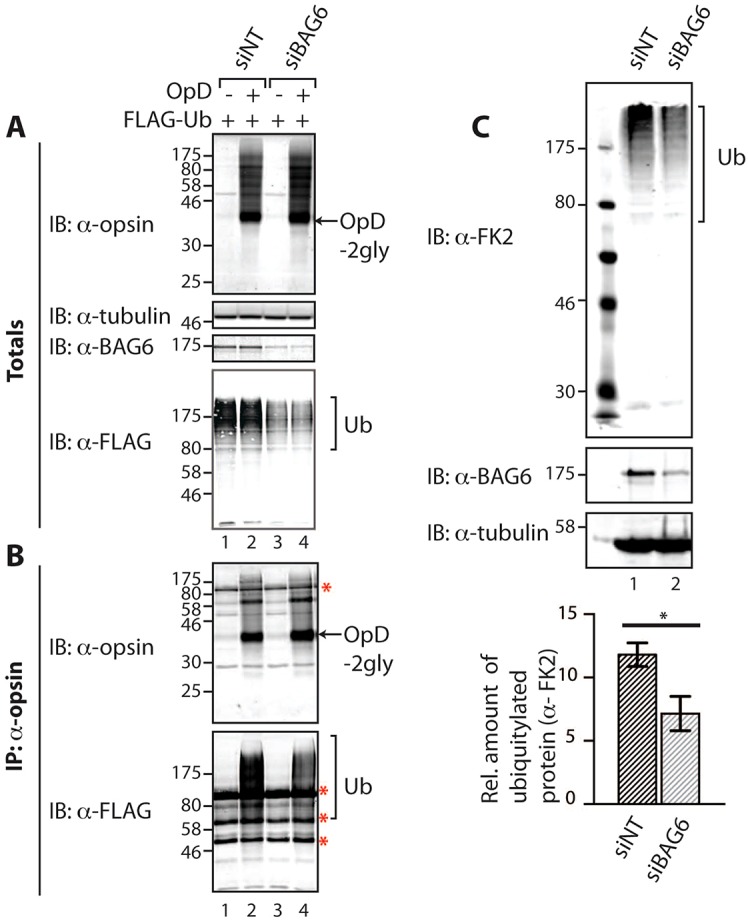
**Endogenous BAG6 modulates polyubiquitylation.** (A,B) The effect of BAG6 knockdown on the ubiquitylation of OpD was analysed. Stable HeLa-OpD cells were transfected with either control non-targeting siRNA (siNT; lanes 1, 2) or BAG6 siRNA (siBAG6; lanes 3, 4) and, 48 hours later, they were induced to express OpD for an additional 16–20 hours. (A) 5% of the samples were kept aside as input controls (totals) and were immunoblotted (IB) for the presence of OpD, endogenous BAG6, tubulin and FLAG−Ub. (B) OpD was immunoprecipitated (IP) with an anti-opsin antibody, and the material recovered was immunoblotted with anti-opsin antibody (to confirm OpD recovery) and with anti-FLAG antibody (to detect the amount of FLAG–Ub that was immunoprecipitated with OpD). Red asterisk, non-related species, as described for Fig. 1D. 2gly, di-glycosylated. (C) Stable HeLa-OpD cells were transfected with either control non-targeting siRNA (siNT, lane 1) or siRNA targeted against BAG6 (siBAG6, lane 2) and, after 48 hours, were induced to express OpD for an additional 16–20 hours. Whole-cell lysates were analysed by immunoblotting with anti-FK2, anti-BAG6 and anti-tubulin antibodies to detect total ubiquitylated species, endogenous BAG6 and tubulin, respectively. The amount of polyubiquitylated protein in lysates prepared from control cells and cells with reduced levels of BAG6 was quantified and normalised to tubulin. Average values obtained for each condition were calculated and plotted as a bar graph. Data show the mean±s.e.m. (three independent repeats); *P<0.05.

To probe the ubiquitylation status of OpD upon the siRNA-mediated reduction of endogenous BAG6, inducible OpD-expressing cells were co-transfected with a plasmid expressing FLAG-tagged ubiquitin. Reducing the levels of the endogenous BAG6 (Fig. 5A, IB: α-BAG6) resulted in a specific reduction in the polyubiquitylation of OpD (Fig. 5B, IB: α-FLAG, cf. lanes 2, 4), consistent with a role for BAG6 in facilitating substrate ubiquitylation. Notably, the BAG6 knockdown also led to a marked reduction in total cellular polyubiquitylated material, as reported by the use of FLAG–ubiquitin (Fig. 5A, IB: α-FLAG). When the effect of the BAG6 knockdown on the amount of total endogenous polyubiquitylated material was analysed, a comparable decrease was observed [Fig. 5C, IB: α-FK2 (an antibody that recognises ubiquitylated proteins)]. Statistical analysis confirmed that the ∼40% reduction in total cellular polyubiquitylated products resulting from a BAG6 knockdown was statistically significant (*P*<0.05; Fig. 5C). We conclude that the loss of BAG6 function following its knockdown results in a reduced level of polyubiquitylated OpD, and our data suggest that BAG6 can also influence the ubiquitylation status of endogenous proteins.

**Fig. 6.**
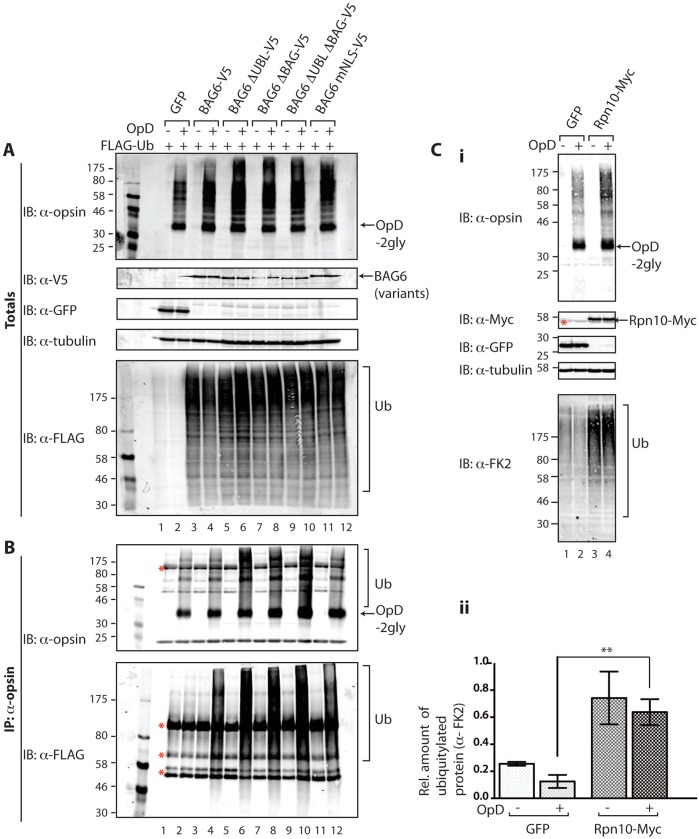
**Exogenous BAG6 and Rpn10 both increase steady-state polyubiquitylation.** (A,B) The polyubiquitylation of total cellular proteins and OpD by BAG6 variants. The effect of overexpressing V5-tagged BAG6 and BAG6 mutants on the polyubiquitylation of total cellular proteins and OpD was analysed. BAG6 proteins, together with FLAG-tagged ubiquitin, were transiently expressed for 24 hours in stable HeLa-OpD cells that were then induced to express OpD for an additional 16–20 hours. (A) 5% of the samples were kept aside as input controls (totals) and immunoblotted (IB) for the presence of OpD, V5-tagged BAG6 proteins, GFP, tubulin and FLAG–Ub. (B) OpD was immunoprecipitated (IP) with an anti-opsin antibody. Immunoprecipitated OpD was immunoblotted with anti-opsin antibody to confirm that immunoprecipitated protein was OpD. Anti-FLAG antibody was used to detect the amount of FLAG–Ub that was covalently bound to OpD. Red asterisks, non-related species, as described for Fig. 1D. (C) Effect of overexpressing Rpn10 on the polyubiquitylation of total cellular proteins. GFP (lanes 1, 2) or Rpn10–Myc (lanes 3, 4) were transiently expressed for 24 hours in stable HeLa-OpD cells that were then either control treated (lanes 1, 3) or induced to express OpD (lanes 2, 4) for an additional 16–20 hours. (i) Whole-cell lysates were prepared and were immunoblotted for OpD, Myc-tagged Rpn10, GFP, tubulin and endogenous ubiquitylated proteins (anti-FK2), as indicated. 2gly, di-glycosylated. (ii) The amount of ubiquitylated protein in lysates prepared from control cells (GFP) and cells expressing exogenous Rpn10–Myc was quantified and normalised to tubulin. Average values obtained for each condition were calculated and plotted as a bar graph. Data show the mean±s.e.m. (three independent repeats); **P<0.01.

Because BAG6 knockdown and BAG6 overexpression both delayed OpD degradation to a similar degree, we investigated the ubiquitylation status of OpD upon the exogenous expression of BAG6. To this end, inducible OpD-expressing cells were co-transfected with plasmids expressing GFP, BAG6–V5 or one of the BAG6 mutants (Fig. 6A, IB α-GFP, lanes 1, 2; α-V5, lanes 3–12) and FLAG-tagged ubiquitin. The effect of BAG6–V5 co-expression on the polyubiquitylation state of OpD was analysed following the specific recovery of the latter by immunoprecipitation. Although comparable levels of OpD were recovered in every case (Fig. 6B, IB: α-opsin, see OpD-2gly), there were clear differences in the amount of FLAG-tagged OpD–ubiquitin conjugates (Fig. 6B, IB: α-FLAG, see Ub). Hence, in control cells, only background levels of polyubiquitylation were apparent in both induced and uninduced cells (Fig. 6B, IB: α-FLAG, cf. lanes 1, 2). By comparison, induction of OpD expression in the presence of BAG6–V5 or any of the mutants tested resulted in increased levels of high-molecular-mass, FLAG–ubiquitin-tagged forms of OpD (Fig. 6B, IB: α-FLAG, cf. lanes 3–12). At a qualitative level, BAG6–V5 and BAG6-mNLS–V5 expression appeared to result in lower levels of polyubiquitylated OpD species than the ΔUBL domain and/or ΔBAG domain mutants (Fig. 6B, cf. lanes 4, 6, 8, 10, 12, see Ub). These data suggest that both the UBL and BAG domains might influence the function(s) of BAG6 at some point prior to the degradation of OpD at the proteasome.

### The exogenous expression of ubiquitin-binding proteins alters steady-state polyubiquitylation

When the effect of BAG6–V5 expression on total polyubiquitylated material was analysed, a striking and reproducible increase in the level of high-molecular-mass polyubiquitylated conjugates was observed, irrespective of OpD expression (Fig. 6A, IB: α-FLAG, cf. lanes 1–4). Thus, the effect of BAG6–V5 expression on steady-state polyubiquitylated species is the opposite of that observed upon a BAG6 knockdown (Fig. 5A,C; Fig. 6A). At a qualitative level, the effects of the various BAG6–V5 mutants on the amount of polyubiquitylated material detected at steady state were comparable (Fig. 6B, IB: α-FLAG, cf. lanes 5–12), ruling out an essential role for either the UBL or BAG domain in this effect. In order to establish whether this effect on total steady-state polyubiquitylated species was specific to BAG6 or simply a consequence of perturbations of ubiquitin homeostasis, we also analysed the effect of overexpressing Rpn10, a well-defined ubiquitin-binding subunit of the proteasome (Wang and Terpstra, 2013). Hence, either GFP or Rpn10–Myc was transiently expressed in the presence or absence of OpD induction (Fig. 6Ci, IB: α-opsin) and total cell lysates were analysed for levels of endogenous ubiquitylated species (Fig. 6Ci, IB: α-FK2). As with BAG6–V5, quantification and statistical analysis confirmed that exogenous Rpn10–Myc expression resulted in a significant increase in the amount of steady-state ubiquitylated material that was independent of OpD expression (Fig. 6Cii). These data suggest that the overexpression of ubiquitin-binding proteins, including both BAG6 and Rpn10, might influence global ubiquitin homeostasis.

## DISCUSSION

### BAG6 promotes OpD degradation

In this study, we demonstrate that BAG6 significantly enhances the degradation and clearance of a model seven-transmembrane-domain ERAD substrate, OpD. OpD interacts directly with BAG6, as shown by its co-immunoprecipitation with both endogenous and exogenously expressed forms of BAG6 (Fig. 1D; Fig. 4C, respectively) and the ability of exogenous BAG6 to cause the non-physiological nuclear relocalisation of OpD (Fig. 3C,D). On this basis, we postulate that BAG6 acts to chaperone retrotranslocated forms of OpD as they enter the cytosol, most likely initially binding to the N-glycosylated form of the protein and maintaining the interaction after subsequent deglycosylation, because we observe both forms of OpD in complex with BAG6.

Notably, although both BAG6 knockdown and BAG6 overexpression delay OpD degradation to a comparable extent, only a reduction in BAG6 levels results in the appearance of OpD-positive inclusions (Fig. 2), suggesting that the mechanistic basis for the two perturbations might be distinct. This hypothesis is further supported by our observation that BAG6 knockdown reduces the steady-state level of polyubiquitylated OpD, whereas BAG6–V5 overexpression increases it (Figs 5, 6). Although the nature of the OpD punctae observed upon BAG6 knockdown remains to be determined, a comparable study by Wang et al., 2011 concluded that a reduction in the levels of endogenous BAG6 resulted in the aggregation of TCRα. Our findings also support the proposal that BAG6 can function as a holdase (Wang et al., 2011), and we speculate that BAG6 contributes to the maintenance of OpD solubility during its retrotranslocation, consistent with a model in which BAG6 plays an important role during or immediately after substrate dislocation from the ER to the cytoplasm (Burr et al., 2013; Claessen and Ploegh, 2011; Wang et al., 2011).

In context of ERAD, BAG6 might be recruited to the ER membrane through its interactions with one or more previously defined ERAD facilitators, including derlin2, p97, gp78, HRD1 and UBXD8 (Claessen and Ploegh, 2011; Wang et al., 2011; Xu et al., 2013). In the case of UBXD8, the UBL domain of BAG6 is known to be required for binding to this protein (Xu et al., 2013), and we find that BAG6 mutants lacking the UBL domain show a substantial reduction in the amount of associated OpD. This behaviour is consistent with inefficient UBXD8-dependent recruitment of the BAG6-ΔUBL and BAG6-ΔUBLΔBAG mutants to the ER membrane to collect retrotranslocating OpD substrates. The loss of the UBL and/or BAG domains does not, however, preclude BAG6 from interacting with OpD, indicating that the central proline-rich region is most likely the principal site for binding hydrophobic substrates, as previously suggested by *in vitro* and *in vivo* studies (Leznicki et al., 2013; Xu et al., 2013). In short, we propose that the UBL but not the BAG domain plays an important role in enabling the efficient loading of ERAD substrates onto the central substrate-binding region of BAG6. Nevertheless, despite the reduction in the amount of OpD associated with the BAG6-ΔUBL mutants, their exogenous expression delayed OpD degradation to a similar extent as the expression of BAG6–V5. Because BAG6 is known to interact with HRD1 in a UBL-independent manner (Wang et al., 2011), we speculate that this delay in OpD degradation might reflect a dominant-negative effect of overexpressed BAG6 through UBL-independent interactions with one or more as-yet-unidentified factors involved in protein quality control and degradation (see below).

### The dominant-negative effect of BAG6 subunit overexpression

The nuclear relocalisation of OpD upon BAG6–V5 expression might suggest that the dominant-negative effect of exogenously expressed BAG6 on OpD degradation occurs through substrate sequestration. However, two of our subsequent observations show this is not the case: firstly, a BAG6 mutant with a defective nuclear localisation signal, BAG6-mNLS–V5, is equally effective at inhibiting OpD degradation; secondly, the efficiency of substrate association with different BAG6 mutants, as judged by co-immunoprecipitation, provides no indication of their effectiveness in perturbing OpD degradation.

In the case of BAG6 subunit overexpression, we propose that the dominant-negative phenotype reflects the normal role of BAG6 as part of a multi-protein complex, the stoichiometry of which is perturbed by the production of exogenous BAG6 (Liu et al., 2014; Prelich, 2012). This hypothesis fits well with a recent study showing that the trimeric BAG6 complex forms higher-order oligomers (Xu et al., 2013). Furthermore, the oligomerisation of the trimeric BAG6 complex is mediated by the central proline-rich region of the BAG6 subunit, which is maintained in all of our BAG6 mutants (Xu et al., 2013). We conclude that an excess of the BAG6 subunit might result in aberrant complex assembly and/or perturb the function of oligomeric BAG6–TRC35–UBL4A trimers at a point downstream of substrate recognition and polyubiquitylation. BAG6 has previously been implicated in the UBL-dependent delivery of ubiquitylated substrates to the Rpn10 subunit of the proteasome (Kikukawa et al., 2005; Minami et al., 2010; Su and Lau, 2009). We hypothesise that exogenous BAG6 subunits might associate with the endogenous BAG6 complex (Xu et al., 2013) and thereby inhibit the delivery of OpD, resulting in reduced levels of substrate deubiquitylation and, hence, degradation (Clague et al., 2012). Notably, the exogenous expression of BAG6 deletion mutants lacking the UBL and/or BAG domains appears to result in a greater increase in the level of polyubiquitylated OpD (Fig. 6B). Hence, both of these domains might contribute to the BAG6-complex-mediated presentation of polyubiquitylated substrates to the proteasome.

### BAG6 influences cellular ubiquitin homeostasis

During the analysis of OpD ubiquitylation we observed a pronounced global effect of a BAG6 knockdown on the level of both total cellular polyubiquitylated species and polyubiquitylated OpD, causing a reduction in both cases (Fig. 5). The exogenous expression of BAG6 had the opposite effect, increasing the steady-state level of both polyubiquitylated OpD and total polyubiquitylated species present in the cell (Fig. 6A,B). Under normal circumstances, ubiquitin levels are tightly regulated, and the ratio of free and conjugated ubiquitin chains is carefully controlled (Kimura and Tanaka, 2010). Our data indicate that BAG6 activity can influence ubiquitin homeostasis, presumably through its effects on the polyubiquitylation status of substrates (Jiang et al., 2011; Minami et al., 2010; Wang et al., 2011; Wu et al., 2012). In order to establish whether this effect on the steady-state level of total cellular polyubiquitylated species was restricted to BAG6, we overexpressed an alternative ubiquitin-binding protein, Rpn10 (Matiuhin et al., 2008; Su and Lau, 2009; Wang and Terpstra, 2013). As for BAG6, the exogenous expression of Rpn10 results in a comparable increase in the level of total polyubiquitylated species that is independent of OpD expression. We therefore conclude that perturbations to the delivery of polyubiquitylated substrates to the proteasome, for example following the overexpression of ubiquitin-binding proteins involved in the delivery of substrates to the proteasome, might result in global changes to cellular ubiquitin homeostasis (cf. Matiuhin et al., 2008). Given the importance of ubiquitin-mediated degradation for a diverse range of cellular activities (Grabbe et al., 2011), this link between BAG6 activity and ubiquitin homeostasis might explain the impact of manipulating BAG6 levels on processes as diverse as cell cycle, apoptosis, antigen presentation and protein degradation (Kawahara et al., 2013). At the level of ERAD-substrate quality control, our data suggest that the BAG6 complex acts to both promote polyubiquitylation and enable the efficient delivery of substrates to the proteasome.

## MATERIALS AND METHODS

### Antibodies and reagents

Mouse anti-opsin antibody that was raised against the N-terminal region of opsin was as described previously (Adamus et al., 1991). Rabbit anti-V5 antibody was made to order (Peptide Speciality Laboratories GmbH). The mouse anti-opsin antibody that recognised the C-terminal region of opsin was from Chemicon. Chicken anti-BAG6, rabbit anti-GFP and rabbit anti-tubulin antibodies were from Abcam. The mouse anti-FLAG (M2 clone) was from Sigma (Poole, UK). The concentrations of primary antibodies used for immunoblotting were as follows; 1∶1000 for anti-opsin and anti-FLAG, 1∶2500 for anti-V5, 1∶5000 for anti-BAG6 and 1∶10,000 for anti-GFP and anti-tubulin. The concentrations of the primary antibodies used for immunofluorescence were 1∶100 for anti-BAG6 and anti-opsin (both the N- and C-terminal-specific antibodies) and 1∶200 for anti-V5. Digitonin and Pansorbin A were obtained from Calbiochem, Protein-A–Sepharose from Genscript and bortezomib from Selleck Chemicals. Protease inhibitor cocktail, V5–agarose, tetracycline and all other chemicals were obtained from Sigma unless stated otherwise.

### DNA plasmid construction and siRNA oligonucleotides

To produce BAG–V5, BAG6 isoform 2 (UniProt ID P46379-2) was cloned into pcDNA 5.0/FRT/-V5 His-TOPO-TA vector (Invitrogen) according to the manufacturer's instructions. BAG6-mNLS–V5 was constructed by site-directed mutagenesis as described previously (Manchen and Hubberstey, 2001). V5-tagged deletion mutants of BAG6 isoform 2, lacking either the UBL domain, the BAG domain or both (BAG6-ΔUBL–V5, BAG6-ΔBAG–V5 or BAG6-ΔUBLΔBAG–V5, respectively), were generated by PCR. BAG6-ΔUBL was amplified with the forward primer 5′-CCTCAGACTCACCTCCCTTCTGGGGCATC-3′ and the reverse primer 5′-GCTGTCAGGCTCCTCCACAGCGGTAC-3′ from BAG6 isoform 2 in pcDNA 5.0-BAG6-V5. BAG6-ΔBAG mutants were amplified with the forward primer 5′-CCCCAGCGCTTCCCCAATGCCCAGC-3 and the reverse primer 5′-CTGGGGGCCCTCACCCTGCATCGTC-3 from pcDNA 5.0-BAG6-V5 and pcDNA 5.0-BAG6-ΔUBL-V5, to generate BAG6-ΔBAG and BAG6-ΔUBLΔBAG, respectively. Plasmids expressing FLAG-tagged ubiquitin (FLAG–Ub), pEGFP-C1 and human Rpn10–Myc were obtained from Sylvie Urbé (University of Liverpool), Martin Lowe (University of Manchester) and Koraljka Husnjak and Ivan Dikic (Goethe University School of Medicine, Frankfurt), respectively. The AllStars control non-targeting siRNA and a previously validated BAG6-targeted siRNA (Winnefeld et al., 2006) (5′-CAGCUCCGGUCUGAUAUACAA-3′) were obtained from Qiagen.

### Mammalian cell culture and transfections

The parental T-REx HeLa cell line was grown in complete DMEM containing 10% fetal bovine serum and 2 mM l-glutamine, and the T-REx HeLa-OpD cell line expressing the opsin degron mutant (Ray-Sinha et al., 2009) under an inducible promoter was grown in the same medium with or without 100 µg/ml hygromycin and 4 µg/ml blasticidin. BAG6 knockdowns were performed with INTERFERin (Polyplus Transfection) according to the manufacturer's protocol, and cells were transfected with 20 nM siRNA. Reductions in the level of the endogenous BAG6 protein were determined by quantitative immunoblotting, essentially as described for OpD (see below). For transient expression of BAG6 or its deletion mutants, cells were transfected with 3∶1 ratios of Lipofectamine 2000 (Invitrogen)∶DNA, according to the manufacturer's protocol. At 24 hours after DNA transfection or 48 hours after siRNA-mediated gene knockdown, T-REx HeLa stable cells were treated with DMEM containing 1 µg/ml tetracycline (Sigma) for an additional 16–20 hours to induce the expression of OpD.

### Cycloheximide chase

T-REx HeLa cells were plated onto a 12-well plate – six wells were plated for time-points 0–5 hours for each condition (i.e. different DNA transfections or siRNA-mediated knockdowns). Cells were transfected with DNA or siRNA as appropriate (see above), induced to express OpD for an additional 16–20 hours and washed twice with PBS. One well for each series was harvested immediately as a ‘zero’ time-point sample, the medium in the remaining wells was replaced with DMEM containing 100 µg/ml cycloheximide (Sigma) and 1 µg/ml tetracycline, and the cells were subsequently harvested at 1-hour intervals. Cells from each well were lysed in 200 µl of SDS sample buffer (0.1 M Tris-HCl pH 6.8, 5 mM EDTA, 0.5 M sucrose, 0.5% l-methionine, 0.01% Bromophenol Blue, 40 mM DTT, 3% SDS), sonicated with five 30-second pulses (BioRaptor) and incubated at 37°C for 1 hour before being analysed by immunoblotting.

### Immunoprecipitation

T-REx HeLa-OpD cells plated on 6-cm dishes were transfected as appropriate and induced to express OpD. The medium was replaced with PBS containing 20 mM *N*-ethylmaleimide (NEM) for 10 minutes, and cells were washed twice with PBS before being scraped into 200 µl of digitonin immunoprecipitation buffer [25 mM Tris-HCl pH 7.4, 150 mM NaCl, 5 mM MgCl_2_, 2% (w/v) digitonin, 1 mM phenylmethanesulfonyl fluoride (PMSF), 2.5 mM NEM and protease inhibitor cocktail]. Cells were solubilised with gentle agitation for 1 hour at 4°C. Lysates were cleared of debris by centrifugation at 3300 ***g*** for 10 minutes at 4°C. 10% of the supernatant was kept aside as total input, and the remaining sample was pre-cleared with 10 µl of Pansorbin A and 10 µl of Protein-A–Sepharose for 1 hour at 4°C. Samples were incubated with either 2 µg of mouse monoclonal anti-opsin antibody (Adamus et al., 1991) bound to 25 µl of Protein-A–Sepharose or 15 µl of anti-V5–agarose for 16–20 hours at 4°C to immunoprecipitate OpD or V5-tagged proteins, respectively. Beads were washed three times with immunoprecipitation buffer supplemented with 0.1% digitonin, and the bound proteins were denatured in 25 µl of SDS sample buffer at 37°C for 1 hour. Samples were then subjected to SDS-PAGE and immunoblot analysis.

### Immunoprecipitation of ubiquitylated protein

T-REx HeLa-OpD cells plated on 6-cm dishes were co-transfected with the DNA of interest and a plasmid expressing FLAG–Ub. The medium was replaced with PBS containing 20 mM NEM for 10 minutes, and the cells were washed twice with PBS before being scraped into 200 µl of 1% radioimmunoprecipitation assay (RIPA) buffer (50 mM Tris-HCl pH 7.5, 150 mM NaCl, 1% SDS, 1% Triton X-100, 1% deoxycholate, 5 mM EDTA) supplemented with 1 mM PMSF, 20 mM NEM and protease inhibitors (Roche) and sonicated with three 30-second pulses. Cells were then solubilised under agitation for 1 hour at 4°C. Lysates were cleared of debris by centrifugation at 13,200 ***g*** for 10 minutes at 4°C. 10% of the supernatant was kept aside as total input, and the remaining sample was diluted with 5× RIPA buffer containing 0.1% SDS and pre-cleared with 10 µl of Pansorbin A and 10 µl of Protein-A–Sepharose for 1 hour at 4°C. OpD was immunoprecipitated by incubating the pre-cleared samples with 4 µl of anti-opsin antibody conjugated to 50 µl of Protein-A–Sepharose for 16–20 hours at 4°C. Beads were washed three times with RIPA buffer containing 0.1% SDS, and bound proteins were denatured in 50 µl of sample buffer at 37°C for 1 hour. Half of each immunoprecipitate was subjected to SDS-PAGE on 12% acrylamide gels, whereas the other half was separated on 8% acrylamide gels to resolve and detect either OpD or polyubiquitylated OpD, respectively. Immunoblot analysis was performed on the resolved samples to detect total and immunoprecipitated polyubiquitylated protein.

### Immunoblotting

Protein samples resolved by SDS-PAGE were transferred onto polyvinylidene fluoride (PVDF) membranes (Millipore) in ice-cold transfer buffer [25 mM Tris-HCl pH 8.3, 192 mM glycine, 1.3 mM SDS, 20% (v/v methanol] at 300 mA constant current for 2 hours. Membranes were blocked in 1× blocking buffer (Sigma) in TBS (10 mM Tris-HCl pH 8.0, 150 mM NaCl) and incubated overnight at 4°C with primary antibodies in 1× blocking buffer-TBS supplemented with 0.1% Tween20 (TBST). Membranes then underwent three 10-minute washes with TBST and were incubated for 1 hour at room temperature with secondary antibodies (anti-rabbit-IgG, anti-mouse-IgG or anti-chicken-IgY forms of IRDye 800 and/or IRDye 680; LI-COR Biosciences, Cambridge, UK) at a dilution of 1∶10,000 in TBST supplemented with 0.01% SDS. Membranes were given three 10-minute washes before being visualised and quantified using the Odyssey Infrared Imaging System (LI-COR Biosciences).

### Quantitative immunoblotting

Following cycloheximide chase and immunoblotting of samples harvested at 0–5-hour time-points, the intensities of OpD-2gly and tubulin were quantified using the Odyssey Image Studio software (LI-COR Biosciences). The amount of OpD present at each time-point was normalised to the tubulin signal obtained for the same time-point, and the resulting OpD values were then expressed relative to the amount of OpD at 0 hours (set at 100%), yielding the percentage of OpD remaining at each time-point. For each experimental condition, the OpD values at each time-point were derived from three independent experiments and then analysed using Prism 4 software (GraphPad) to calculate the mean±s.e.m. Mean values and error bars at each time-point were plotted along with a curve of best fit, enabling an estimate of the half-life of OpD under each experimental condition.

### Immunofluorescence microscopy

Cells grown on coverslips were transfected with either DNA or siRNA and/or treated as described and then fixed with 4% paraformaldehyde for 10–15 minutes. Free aldehyde groups were quenched with 0.1 M glycine, followed by permeabilisation of the cell membranes with 0.1% Triton X-100 and 0.05% SDS in PBS for 5 minutes at room temperature. Cells were then stained with primary antibodies for 30 minutes, washed and incubated with the corresponding Alexa-Fluor-488- or Alexa-Fluor-594-conjugated secondary antibodies (Molecular Probes, Invitrogen) used at 1∶1000 concentrations for another 30 minutes. Coverslips were mounted on glass slides and viewed using an Olympus BX60 upright microscope attached to a CoolSnap ES camera (Roper Scientific). Imaging was performed with the Metamorph software (Universal Imaging Corporation). Images were processed using ImageJ and Adobe Illustrator CS4.

### Data and statistical analysis

The antibody labelling of proteins transferred to immunoblots was quantified using Odyssey Image Studio software (LI-COR Biosciences). The resulting data were processed using the Prism 4 software (GraphPad) and presented as the mean±s.e.m. Where appropriate, two-tailed *t*-tests were performed to determine *P*-values for paired samples. A value of *P*<0.05 (*) or less [*P*<0.01 (**)] was considered statistically significant.

## Supplementary Material

Supplementary Material

## References

[b1] Adamus G. Zam Z. S. Arendt A. Palczewski K. McDowell J. H. Hargrave P. A. (1991). Anti-rhodopsin monoclonal antibodies of defined specificity: characterization and application. Vision Res. 31, 17–31 10.1016/0042--6989(91)90069--H 2006550

[b2] Akahane T. Sahara K. Yashiroda H. Tanaka K. Murata S. (2013). Involvement of Bag6 and the TRC pathway in proteasome assembly. Nat. Commun. 4, 2234 10.1038/ncomms3234 23900548

[b3] Buchberger A. Bukau B. Sommer T. (2010). Protein quality control in the cytosol and the endoplasmic reticulum: brothers in arms. Mol. Cell 40, 238–252 10.1016/j.molcel.2010.10.001 20965419

[b4] Burr M. L. van den Boomen D. J. H. Bye H. Antrobus R. Wiertz E. J. Lehner P. J. (2013). MHC class I molecules are preferentially ubiquitinated on endoplasmic reticulum luminal residues during HRD1 ubiquitin E3 ligase-mediated dislocation. Proc. Natl. Acad. Sci. USA 110, 14290–14295 10.1073/pnas.1303380110 23929775 PMC3761574

[b5] Claessen J. H. L. Ploegh H. L. (2011). BAT3 guides misfolded glycoproteins out of the endoplasmic reticulum. PLoS ONE 6, e28542 10.1371/journal.pone.0028542 22174835 PMC3234288

[b6] Claessen J. H. Kundrat L. Ploegh H. L. (2012). Protein quality control in the ER: balancing the ubiquitin checkbook. Trends Cell Biol. 22, 22–32 10.1016/j.tcb.2011.09.010 22055166 PMC3564647

[b7] Clague M. J. Coulson J. M. Urbé S. (2012). Cellular functions of the DUBs. J. Cell Sci. 125, 277–286 10.1242/jcs.090985 22357969

[b8] Ernst R. Claessen J. H. L. Mueller B. Sanyal S. Spooner E. van der Veen A. G. Kirak O. Schlieker C. D. Weihofen W. A. Ploegh H. L. (2011). Enzymatic blockade of the ubiquitin-proteasome pathway. PLoS Biol. 8, e1000605 10.1371/journal.pbio.1000605 21468303 PMC3066133

[b9] Fleig L. Bergbold N. Sahasrabudhe P. Geiger B. Kaltak L. Lemberg M. K. (2012). Ubiquitin-dependent intramembrane rhomboid protease promotes ERAD of membrane proteins. Mol. Cell 47, 558–569 10.1016/j.molcel.2012.06.008 22795130

[b10] Ghaemmaghami S. Huh W-K. Bower K. Howson R. W. Belle A. Dephoure N. O'Shea E. K. Weissman J. S. (2003). Global analysis of protein expression in yeast. Nature 425, 737–741 10.1038/nature02046 14562106

[b11] Goeckeler J. L. Brodsky J. L. (2010). Molecular chaperones and substrate ubiquitination control the efficiency of endoplasmic reticulum-associated degradation. Diabetes Obes. Metab. 12, Suppl. 2 32–38 10.1111/j.1463--1326.2010.01273.x 21029298 PMC3071497

[b12] Grabbe C. Husnjak K. Dikic I. (2011). The spatial and temporal organization of ubiquitin networks. Nat. Rev. Mol. Cell Biol. 12, 295–307 10.1038/nrm3099 21448225 PMC3654194

[b13] Hessa T. Sharma A. Mariappan M. Eshleman H. D. Gutierrez E. Hegde R. S. (2011). Protein targeting and degradation are coupled for elimination of mislocalized proteins. Nature 475, 394–397 10.1038/nature10181 21743475 PMC3150218

[b14] Jiang W. Wang S. Xiao M. Lin Y. Zhou L. Lei Q. Xiong Y. Guan K-L. Zhao S. (2011). Acetylation regulates gluconeogenesis by promoting PEPCK1 degradation via recruiting the UBR5 ubiquitin ligase. Mol. Cell 43, 33–44 10.1016/j.molcel.2011.04.028 21726808 PMC3962309

[b15] Jonikas M. C. M. Collins S. R. S. Denic V. Oh E. Quan E. M. E. Schmid V. Weibezahn J. Schwappach B. Walter P. Weissman J. S. J. (2009). Comprehensive characterization of genes required for protein folding in the endoplasmic reticulum. Science 323, 1693–1697 10.1126/science.1167983 19325107 PMC2877488

[b16] Kawahara H. Minami R. Yokota N. (2013). BAG6/BAT3: emerging roles in quality control for nascent polypeptides. J. Biochem. 153, 147–160 23275523 10.1093/jb/mvs149

[b17] Kikukawa Y. Minami R. Shimada M. Kobayashi M. Tanaka K. Yokosawa H. Kawahara H. (2005). Unique proteasome subunit Xrpn10c is a specific receptor for the antiapoptotic ubiquitin-like protein Scythe. FEBS J. 272, 6373–6386 10.1111/j.1742--4658.2005.05032.x 16336274

[b18] Kimura Y. Tanaka K. (2010). Regulatory mechanisms involved in the control of ubiquitin homeostasis. J. Biochem. 147, 793–798 10.1093/jb/mvq044 20418328

[b19] Krenciute G. Liu S. Yucer N. Shi Y. Ortiz P. Liu Q. Kim B. J. Odejimi A. O. Leng M. Qin J. (2013). Nuclear BAG6-UBL4A-GET4 complex mediates DNA damage signaling and cell death. J. Biol. Chem. 288, 20547–20557 10.1074/jbc.M112.443416 23723067 PMC3711319

[b20] Lee J-G. Ye Y. (2013). Bag6/Bat3/Scythe: a novel chaperone activity with diverse regulatory functions in protein biogenesis and degradation. Bioessays 35, 377–385 10.1002/bies.201200159 23417671 PMC9379985

[b21] Leznicki P. High S. (2012). SGTA antagonizes BAG6-mediated protein triage. Proc. Natl. Acad. Sci. USA 109, 19214–19219 10.1073/pnas.1209997109 23129660 PMC3511132

[b22] Leznicki P. Clancy A. Schwappach B. High S. (2010). Bat3 promotes the membrane integration of tail-anchored proteins. J. Cell Sci. 123, 2170–2178 10.1242/jcs.066738 20516149 PMC2886740

[b23] Leznicki P. Roebuck Q. P. Wunderley L. Clancy A. Krysztofinska E. M. Isaacson R. L. Warwicker J. Schwappach B. High S. (2013). The association of BAG6 with SGTA and tail-anchored proteins. PLoS ONE 8, e59590 10.1371/journal.pone.0059590 23533635 PMC3606182

[b24] Liu Y. Soetandyo N. Lee J-G. Liu L. Xu Y. Clemons W. M. Jr and Ye Y. (2014). USP13 antagonizes gp78 to maintain functionality of a chaperone in ER-associated degradation. Elife 3, e01369 10.7554/eLife.01369 24424410 PMC3889402

[b25] Lynes E. M. Simmen T. (2011). Urban planning of the endoplasmic reticulum (ER): how diverse mechanisms segregate the many functions of the ER. Biochim. Biophys. Acta 1813, 1893–1905 10.1016/j.bbamcr.2011.06.011 21756943 PMC7172674

[b26] Manchen S. T. Hubberstey A. V. (2001). Human Scythe contains a functional nuclear localization sequence and remains in the nucleus during staurosporine-induced apoptosis. Biochem. Biophys. Res. Commun. 287, 1075–1082 10.1006/bbrc.2001.5701 11587531

[b27] Matiuhin Y. Kirkpatrick D. S. Ziv I. Kim W. Dakshinamurthy A. Kleifeld O. Gygi S. P. Reis N. Glickman M. H. (2008). Extraproteasomal Rpn10 restricts access of the polyubiquitin-binding protein Dsk2 to proteasome. Mol. Cell 32, 415–425 10.1016/j.molcel.2008.10.011 18995839 PMC2643056

[b28] Minami R. Hayakawa A. Kagawa H. Yanagi Y. Yokosawa H. Kawahara H. (2010). BAG-6 is essential for selective elimination of defective proteasomal substrates. J. Cell Biol. 190, 637–650 10.1083/jcb.200908092 20713601 PMC2928017

[b29] Prelich G. (2012). Gene overexpression: uses, mechanisms, and interpretation. Genetics 190, 841–854 10.1534/genetics.111.136911 22419077 PMC3296252

[b30] Ray-Sinha A. Cross B. C. Mironov A. Wiertz E. High S. (2009). Endoplasmic reticulum-associated degradation of a degron-containing polytopic membrane protein. Mol. Membr. Biol. 26, 448–464 10.3109/09687680903333839 19878048 PMC3428838

[b31] Su V. Lau A. F. (2009). Ubiquitin-like and ubiquitin-associated domain proteins: significance in proteasomal degradation. Cell. Mol. Life Sci. 66, 2819–2833 10.1007/s00018--009--0048--9 19468686 PMC2725189

[b32] Wang X. Terpstra E. J. M. (2013). Ubiquitin receptors and protein quality control. J. Mol. Cell. Cardiol. 55, 73–84 10.1016/j.yjmcc.2012.09.012 23046644 PMC3571097

[b33] Wang Q. Liu Y. Soetandyo N. Baek K. Hegde R. Ye Y. (2011). A ubiquitin ligase-associated chaperone holdase maintains polypeptides in soluble states for proteasome degradation. Mol. Cell 42, 758–770 10.1016/j.molcel.2011.05.010 21636303 PMC3138499

[b34] Wiertz E. J. Jones T. R. Sun L. Bogyo M. Geuze H. J. Ploegh H. L. (1996a). The human cytomegalovirus US11 gene product dislocates MHC class I heavy chains from the endoplasmic reticulum to the cytosol. Cell 84, 769–779 10.1016/S0092--8674(00)81054--5 8625414

[b35] Wiertz E. J. Tortorella D. Bogyo M. Yu J. Mothes W. Jones T. R. Rapoport T. A. Ploegh H. L. (1996b). Sec61-mediated transfer of a membrane protein from the endoplasmic reticulum to the proteasome for destruction. Nature 384, 432–438 10.1038/384432a0 8945469

[b36] Winnefeld M. Grewenig A. Schnölzer M. Spring H. Knoch T. A. Gan E. C. Rommelaere J. Cziepluch C. (2006). Human SGT interacts with Bag-6/Bat-3/Scythe and cells with reduced levels of either protein display persistence of few misaligned chromosomes and mitotic arrest. Exp. Cell Res. 312, 2500–2514 10.1016/j.yexcr.2006.04.020 16777091

[b37] Wu W. Song W. Li S. Ouyang S. Fok K. L. Diao R. Miao S. Chan H. C. Wang L. (2012). Regulation of apoptosis by Bat3-enhanced YWK-II/APLP2 protein stability. J. Cell Sci. 125, 4219–4229 10.1242/jcs.086553 22641691

[b38] Xu Y. Cai M. Yang Y. Huang L. Ye Y. (2012). SGTA recognizes a noncanonical ubiquitin-like domain in the Bag6-Ubl4A-Trc35 complex to promote endoplasmic reticulum-associated degradation. Cell Rep. 2, 1633–1644 10.1016/j.celrep.2012.11.010 23246001 PMC3534891

[b39] Xu Y. Liu Y. Lee J-G. Ye Y. (2013). A ubiquitin-like domain recruits an oligomeric chaperone to a retrotranslocation complex in endoplasmic reticulum-associated degradation. J. Biol. Chem. 288, 18068–18076 10.1074/jbc.M112.449199 23665563 PMC3689951

[b40] Yewdell J. W. Lacsina J. R. Rechsteiner M. C. Nicchitta C. V. (2011). Out with the old, in with the new? Comparing methods for measuring protein degradation. Cell Biol. Int. 35, 457–462 10.1042/CBI20110055 21476986 PMC3727619

[b41] Yong S. T. Wang X-F. (2012). A novel, non-apoptotic role for Scythe/BAT3: a functional switch between the pro- and anti-proliferative roles of p21 during the cell cycle. PLoS ONE 7, e38085 10.1371/journal.pone.0038085 22761665 PMC3384656

