# Supplementary material for: BAG6 regulates the quality control of a polytopic ERAD substrate
Source: J Cell Sci. 2014 Jul 1;127(13):2898–909. doi: 10.1242/jcs.145565 (PMC4075357; doi:10.1242/jcs.145565)
Supplement: Supplementary Material [file supp_127_13_2898__index.html]

BAG6 regulates the quality control of a polytopic ERAD substrate — Supplementary Material 

# BAG6 regulates the quality control of a polytopic ERAD substrate

## JCS145565 Supplementary Material

**Files in this Data Supplement:**

- **Supplementary Material**
