# Supplementary material for: BAG6 regulates the quality control of a polytopic ERAD substrate
Source: J Cell Sci. 2014 Jul 1;127(13):2898–909. doi: 10.1242/jcs.145565 (PMC4075357; doi:10.1242/jcs.145565)
Supplement: Supplementary Material [file supp_127.13.2898_JCS145565.pdf]

Supplementary Figures

Figure S1. (A-C) Proteasomal inhibition inhibits OpD degradation. (A-B) OpD HeLa cells were induced to express OpD for 16-20 hours followed by a 5-hour treatment with either 0.1% v/v DMSO or 10 μM bortezomib (BZ). **(A)** Degradation of OpD in the presence or absence BZ was compared by immunoblot analysis of whole cell lysates prepared at 0 hours and 1-hour time intervals during a 5-hour long cycloheximide chase. **(B)** OpD levels were quantified at each time-point and the resulting data analysed as described in the Materials and Methods section of the main text. Mean OpD levels and errors (-/+ s.e.m.) were calculated from at least three independent repeats and plotted along with the curves of best fit for each condition. **(C)** Effect of over-expressing GFP or BAG6-V5 on OpD degradation was compared to the effect of DMSO and BZ treatments: data from Fig. 3B (main text) and supplementary Fig. S1B were combined to allow direct comparison. **(D) The effect of BAG6-V5 expression on the steady state level of OpD is titratable.** OpD HeLa cells were transfected with increasing amounts of BAG6-V5- 0 μg (lane 1), 0.5 μg (lane 2), 1 μg (lane 3), 1.5 μg (lane 4) and 2 μg (lane 5) for 24 hours, followed by induction of OpD for an additional 16-20 hours before whole cell lysates were prepared and analysed by immunoblotting with anti-opsin, anti-V5 and anti-βactin antibodies. A high exposure of the anti-opsin immunoblot is included (middle panel) to show an increase in the unglycosylated OpD signal (OpD-0gly) observed at higher expression levels of BAG6-V5.

Figure S2. BAG6-mNLS delays OpD degradation. (A) Effect of BAG6 mNLS-V5 over-expression on OpD was visualised by immunofluorescence microscopy. OpD HeLa cells transiently expressing BAG6 mNLS-V5 for 24 hours were induced to express OpD for an additional 16-20 hours before being fixed and stained with an anti-opsin (N-terminal) antibody to detect OpD (red) and anti-V5 antibody to detect over-expressed BAG6 mNLS (green). Scale bar, 10 μm. **(B and C)** A cycloheximide chase was performed with OpD HeLa cells that were transiently expressing BAG6-V5 or BAG6 mNLS-V5 for 24 hours prior to the induction of OpD for a further 16-20 hours **(B)** Whole cell lysates from each time point were immunoblotted for OpD (IB: α-opsin), BAG6-V5 variants (IB: α-V5) and tubulin (IB: α-tubulin). **(C)** OpD degradation in the presence of either BAG6-V5 or BAG6 mNLS-V5 was compared. The amounts of opsin at 0 hours and each subsequent 1-hour time intervals were quantified and the resulting data analysed as described in the Materials and Methods section of the main text.

Mean OpD levels and errors (-/+ s.e.m.) were calculated from at least three independent repeats and plotted along with the curves of best fit for each condition.

Figure S3. BAG6 and OpD do not interact post-lysis. The OpD HeLa cell line was either induced to express OpD (lanes 1 and 5) or transfected with BAG6-V5 (lanes 2 and 6) for 24 hours. Lysates were prepared from these two sets of cells using digitonin containing buffer (see Materials and Methods section of main text), a subset of samples were mixed (lane 3 = lane 1 + 2, lanes 7= lane 5 +6) and a parallel set of mixed samples processed by immunoprecipitation of OpD (lane 4) or BAG6-V5 (lane 8). Both total extracts (lanes 1 to 3 and 5 to 7), and immunoprecipitated products were analysed by immunoblotting for OpD and V5-tagged BAG6. This analysis showed no significant co-immunoprecipitation of BAG6-V5 with OpD (lanes 4, IB: α-BAG6), nor was any OpD detectable in the products recovered with BAG6-V5 (lane 8, IB: α-opsin). A number of non-related species were observed in the samples subjected to immunoprecipitation (see red asterisks), these are most likely due to recognition of the primary antibodies used during immunoisolation.

Figure S4. BAG6-V5 deletion mutants all delay OpD degradation to a comparable degree. A cycloheximide chase was performed on the OpD HeLa cell line that was made to transiently express either BAG6-V5/ BAG6ΔUBL-V5/ BAG6 ΔBAG-V5/ BAG6 ΔUBLΔBAG-V5 for 24 hours followed by induction of OpD for 16-20 hours, in order to analyse the degradation of OpD under these different conditions. **(A)** **(i)** OpD was induced in the presence of BAG6-V5 (lanes 1-6) or BAG6ΔUBL-V5 (lanes 7-12) expression . Samples from each time-point were analysed by immunoblotting with anti-opsin, anti-V5 and anti-tubulin antibodies. **(ii)** Amounts of OpD at each time-point were quantified and statistically analysed as described in Materials and medthods. Mean and error bars values (-/+ s.e.m.) were calculated from at least three independent repeats and plotted along with the curves of best fit for each condition. **(B and C)** Identical to **A,** but for OpD in the presence of BAG6 ΔBAG-V5 (Bi: lanes 7-12) or BAG6 ΔUBLΔBAG-V5 (Ci: lanes 7-12), respectively.

Figure S1

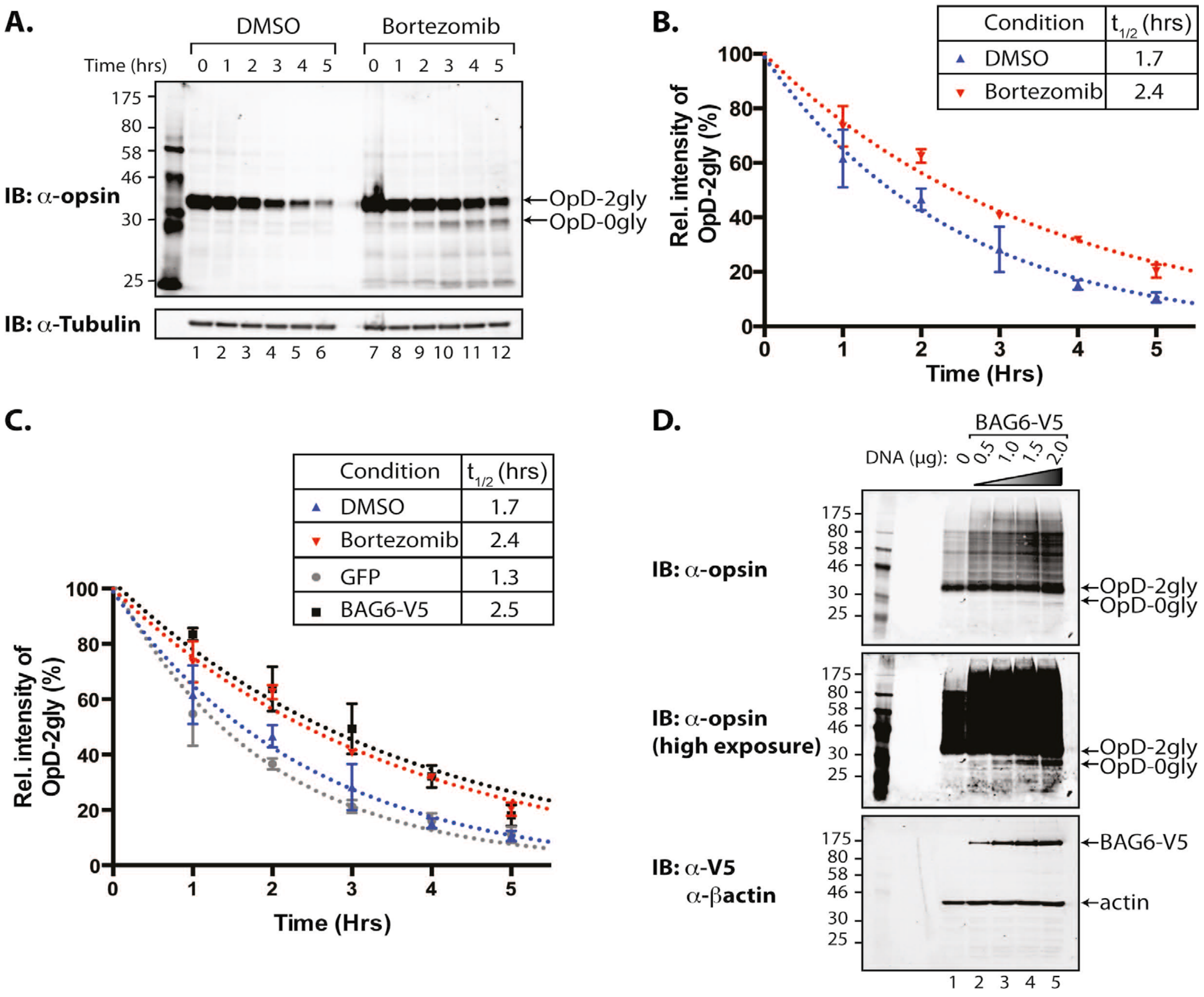

A.
DMSO
Bortezomib
Time (hrs)
0 1 2 3 4 5
0 1 2 3 4 5
175
80
58
46
30
25
IB: α-opsin
←OpD-2gly
←OpD-0gly
IB: α-Tubulin
1 2 3 4 5 6
7 8 9 10 11 12
B.
Condition
t1/2 (hrs)
DMSO
1.7
Bortezomib
2.4
Rel. intensity of OpD-2gly (%)
Time (Hrs)
C.
Condition
t1/2 (hrs)
DMSO
1.7
Bortezomib
2.4
GFP
1.3
BAG6-V5
2.5
Rel. intensity of OpD-2gly (%)
Time (Hrs)
D.
BAG6-V5
DNA (μg):
0
0.5
1.0
1.5
2.0
IB: α-opsin
←OpD-2gly
←OpD-0gly
IB: α-opsin (high exposure)
←OpD-2gly
←OpD-0gly
IB: α-V5
α-βactin
←BAG6-V5
←actin
1 2 3 4 5

Figure S2

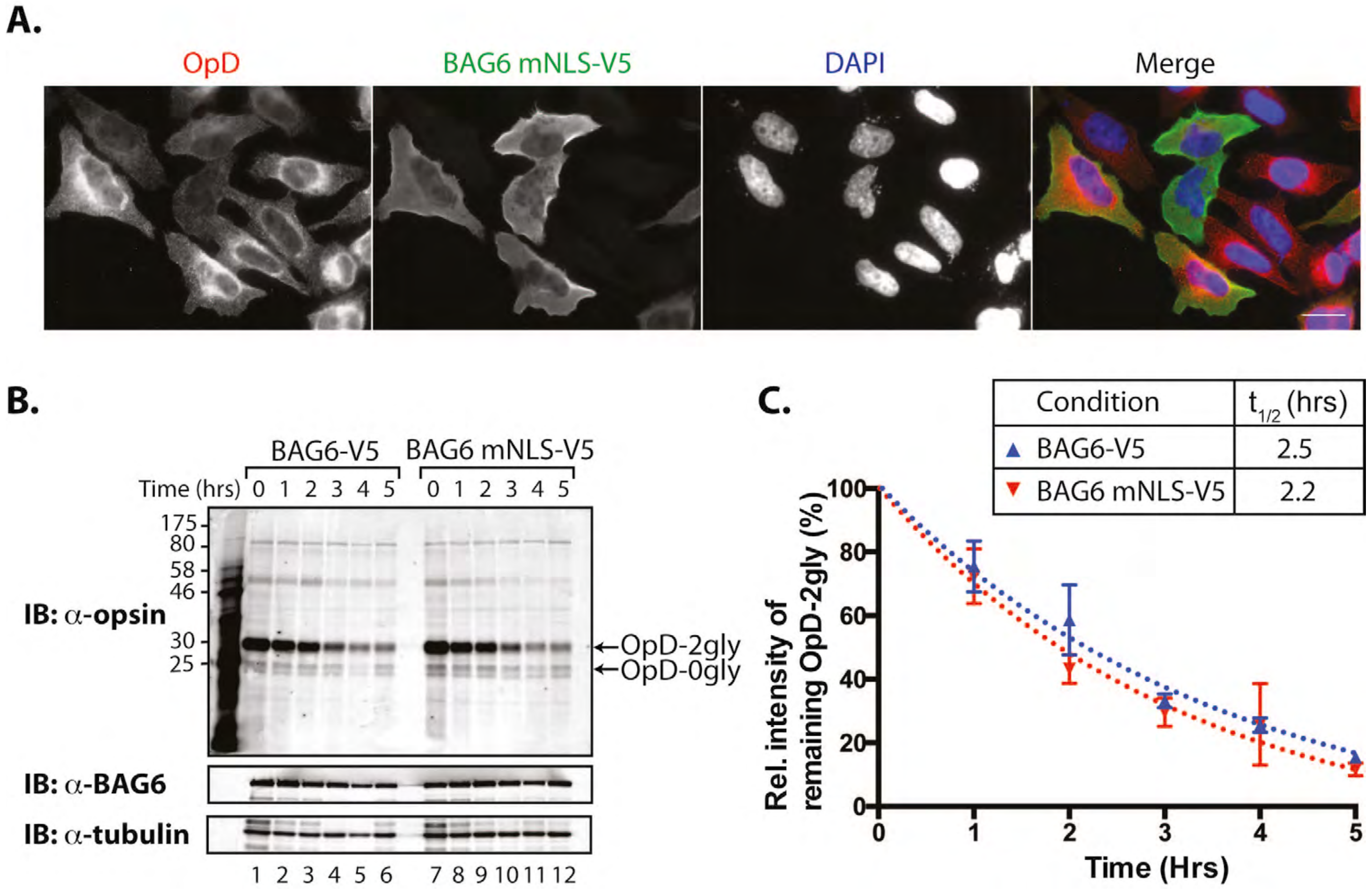
A.
OpD
BAG6 mNLS-V5
DAPI
Merge
B.
BAG6-V5
BAG6 mNLS-V5
Time (hrs) 0 1 2 3 4 5 0 1 2 3 4 5
175
80
58
46
30
25
IB: α-opsin
←OpD-2gly
←OpD-0gly
IB: α-BAG6
IB: α-tubulin
1 2 3 4 5 6 7 8 9 10 11 12
C.
Condition
t1/2 (hrs)
BAG6-V5 2.5
BAG6 mNLS-V5 2.2
Rel. intensity of remaining OpD-2gly (%)
100
80
60
40
20
0
0 1 2 3 4 5
Time (Hrs)

Figure S3

A.

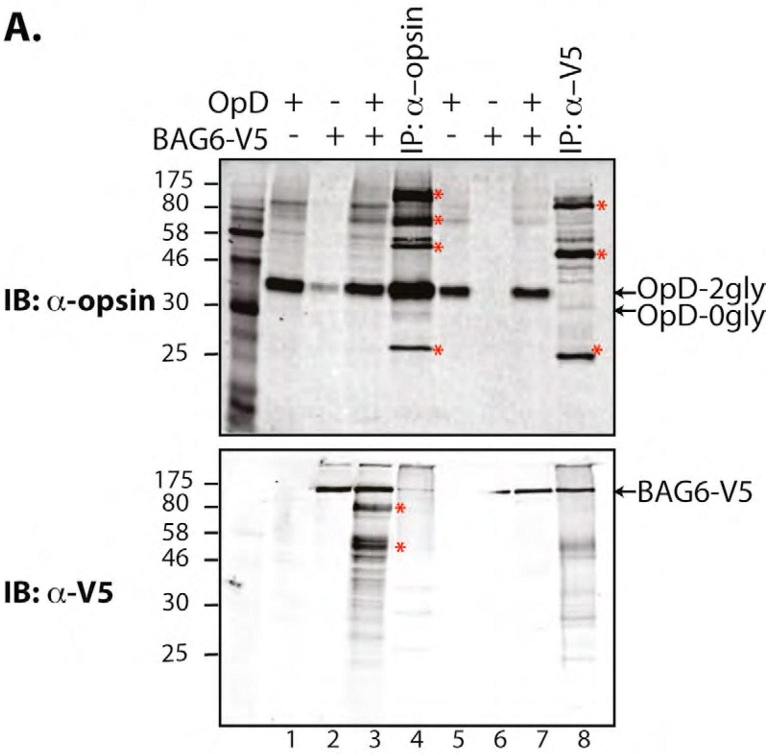

OpD + - + IP: α–opsin + - + IP: α–V5
BAG6-V5 - + + - + +
IB: α-opsin
175
80
58
46
30
25
OpD-2gly
OpD-0gly
IB: α-V5
175
80
58
46
30
25
BAG6-V5
1 2 3 4 5 6 7 8

Figure S4

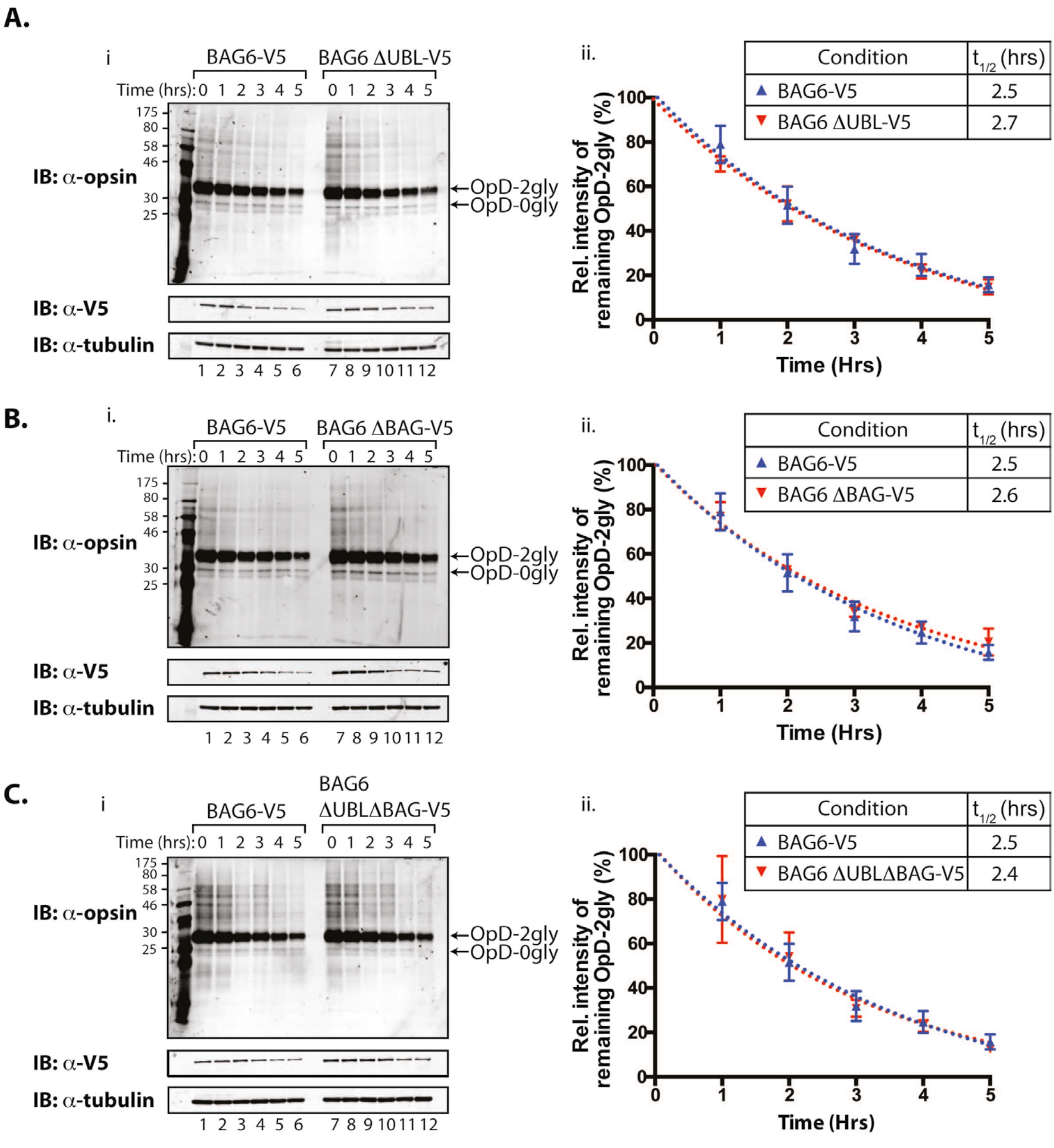
A.
i
BAG6-V5
BAG6 ΔUBL-V5
Time (hrs): 0 1 2 3 4 5 0 1 2 3 4 5
175
80
58
46
30
25
IB: α-opsin
←OpD-2gly
←OpD-0gly
IB: α-V5
IB: α-tubulin
1 2 3 4 5 6 7 8 9 10 11 12
ii.
Condition
t1/2 (hrs)
BAG6-V5 2.5
BAG6 ΔUBL-V5 2.7
Rel. intensity of remaining OpD-2gly (%)
Time (Hrs)
B.
i.
BAG6-V5
BAG6 ΔBAG-V5
Time (hrs): 0 1 2 3 4 5 0 1 2 3 4 5
IB: α-opsin
←OpD-2gly
←OpD-0gly
IB: α-V5
IB: α-tubulin
1 2 3 4 5 6 7 8 9 10 11 12
ii.
Condition
t1/2 (hrs)
BAG6-V5 2.5
BAG6 ΔBAG-V5 2.6
Rel. intensity of remaining OpD-2gly (%)
Time (Hrs)
C.
i
BAG6-V5
BAG6 ΔUBLΔBAG-V5
Time (hrs): 0 1 2 3 4 5 0 1 2 3 4 5
IB: α-opsin
←OpD-2gly
←OpD-0gly
IB: α-V5
IB: α-tubulin
1 2 3 4 5 6 7 8 9 10 11 12
ii.
Condition
t1/2 (hrs)
BAG6-V5 2.5
BAG6 ΔUBLΔBAG-V5 2.4
Rel. intensity of remaining OpD-2gly (%)
Time (Hrs)
